# Artificial Sweeteners in a Large Canadian River Reflect Human Consumption in the Watershed

**DOI:** 10.1371/journal.pone.0082706

**Published:** 2013-12-11

**Authors:** John Spoelstra, Sherry L. Schiff, Susan J. Brown

**Affiliations:** 1 Water Science and Technology Directorate, Environment Canada, Burlington, Ontario, Canada; 2 Department of Earth and Environmental Sciences, University of Waterloo, Waterloo, Ontario, Canada; University of Yamanashi, Japan

## Abstract

Artificial sweeteners have been widely incorporated in human food products for aid in weight loss regimes, dental health protection and dietary control of diabetes. Some of these widely used compounds can pass non-degraded through wastewater treatment systems and are subsequently discharged to groundwater and surface waters. Measurements of artificial sweeteners in rivers used for drinking water production are scarce. In order to determine the riverine concentrations of artificial sweeteners and their usefulness as a tracer of wastewater at the scale of an entire watershed, we analyzed samples from 23 sites along the entire length of the Grand River, a large river in Southern Ontario, Canada, that is impacted by agricultural activities and urban centres. Municipal water from household taps was also sampled from several cities within the Grand River Watershed. Cyclamate, saccharin, sucralose, and acesulfame were found in elevated concentrations despite high rates of biological activity, large daily cycles in dissolved oxygen and shallow river depth. The maximum concentrations that we measured for sucralose (21 µg/L), cyclamate (0.88 µg/L), and saccharin (7.2 µg/L) are the highest reported concentrations of these compounds in surface waters to date anywhere in the world. Acesulfame persists at concentrations that are up to several orders of magnitude above the detection limit over a distance of 300 km and it behaves conservatively in the river, recording the wastewater contribution from the cumulative population in the basin. Acesulfame is a reliable wastewater effluent tracer in rivers. Furthermore, it can be used to assess rates of nutrient assimilation, track wastewater plume dilution, separate human and animal waste contributions and determine the relative persistence of emerging contaminants in impacted watersheds where multiple sources confound the usefulness of other tracers. The effects of artificial sweeteners on aquatic biota in rivers and in the downstream Great Lakes are largely unknown.

## Introduction

Artificial sweeteners (AS) are increasingly used as a sugar substitute to reduce caloric intake, for dental health protection and for control of diabetes. As a result of the use of artificial sweeteners in food and beverages and the ability of some of these compounds to pass non-degraded through wastewater treatment plants (WWTPs), AS are being detected in rivers and lakes (e.g. [1]–[4]) and groundwater (e.g. [4]–[7]) around the world. Some AS are also able to pass through water treatment plants and are subsequently found in municipal potable water supplies (e.g. [2], [9], [10]), typically where the source water intake for one municipality is downstream from the WWTP discharge of another.

One AS, acesulfame, is particularly resistant to degradation in WWTPs and has been proposed as an ideal tracer of wastewater in the environment (e.g. [2]). Acesulfame has been shown to be mobile and recalcitrant in groundwater [2], [5], [6], making it a suitable wastewater tracer in the subsurface as well as surface water bodies. Additionally, acesulfame removal by the various processes used in water treatment plants for the production of municipal potable water has been shown to be incomplete [9]. To investigate the concentrations and behavior of AS at the scale of an entire watershed, we conducted a study of the Grand River in Ontario, Canada.

**Figure 1:**
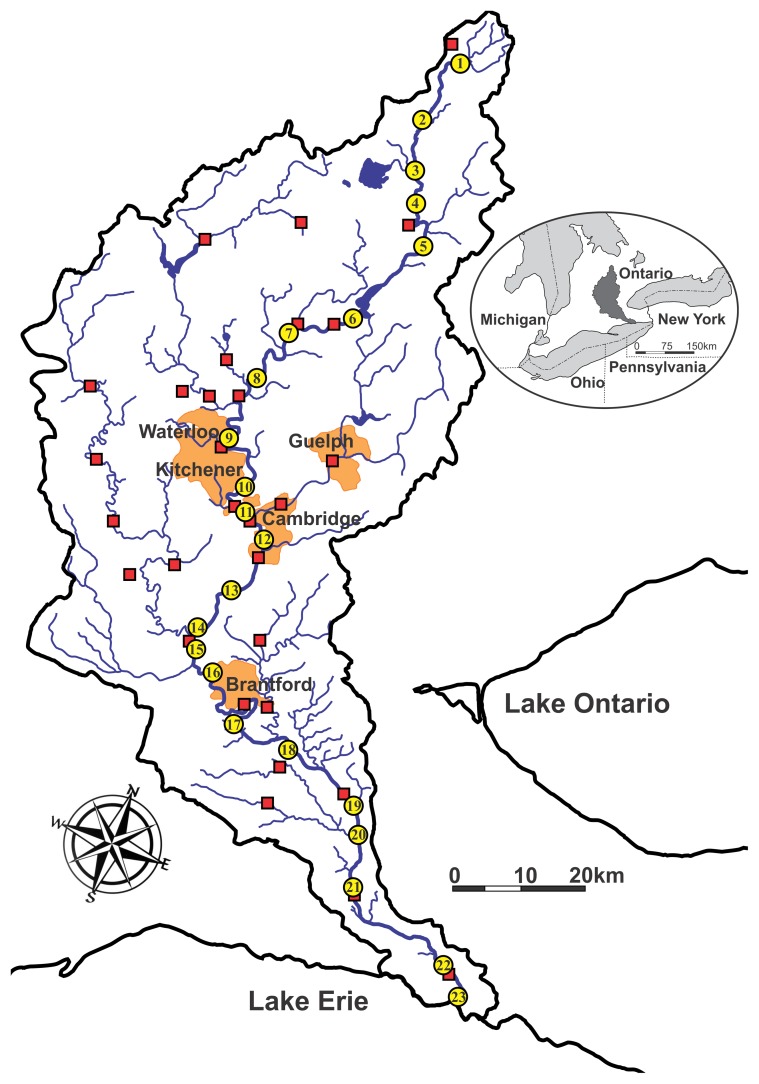
Grand River Watershed (6,800 km^2^), Ontario, Canada. Numbered circles and red squares indicate the 23 sampling sites and 30 WWTPs, respectively. Site numbering starts in the headwaters and increases downstream, terminating at Port Maitland where the Grand River discharges to Lake Erie. The 3 largest WWTPs by rated capacity are Kitchener, Brantford and Waterloo.

The Grand River Watershed in southern Ontario contains the largest Canadian river discharging to Lake Erie, one of the Great Lakes bordered by Canada and the United States (Fig.1). Predominant land use in the watershed is agricultural (over 80%) but a large urban population (>600,000) is concentrated in the central portion of the watershed. A total of 30 WWTPs servicing a current population of approximately 800,000, discharge to the Grand River and its tributaries. The total population of the Grand River Watershed is currently 960,000 and expected to increase to over 1,400,000 by 2041 [11], with most of this increase occurring in urban areas on sewers. Currently, over 500,000 people rely on Grand River water for domestic use either directly after treatment (>120,000 people) or via groundwater recharge schemes. Therefore the ability of the Grand River to function both as a diluter/assimilator of waste and as a safe source of raw water for drinking water production, while conserving ecological health, is of vital concern.

Analysis of artificial sweeteners (AS) presents an extremely powerful tool for tracing the impact of wastewater constituents on receiving waters. Nutrient loads to the Grand River are high due to intensifying agricultural activity and WWTPs, leading to high rates of river metabolism, large daily cycles in dissolved oxygen (DO) [12] and summer nighttime hypoxia below the largest WWTP (Kitchener). River discharge is heavily regulated by reservoirs used for flood prevention and low flow augmentation. To assess the load and persistence of AS in a highly impacted river, we conducted a longitudinal survey by selecting 23 sites over the 300 km river length, starting at the upper 2^nd^ order reach and ending at the discharge point to Lake Erie where the river is 7^th^ order (Fig. 1).

## Methods

Samples were collected along the length of the Grand River at 23 sites from headwaters to mouth (Fig.1). No specific permissions were required for these sampling locations since they were all publically accessible sites. In the upper one third of the watershed (sites 1–9), the surficial geology is characterized by glacial till. The urban reach near the center of the watershed (sites 10–12) is followed by a zone of higher groundwater recharge (sites 13–16) due to the presence of glacial moraines. The lower third of the watershed (sites 17–23) is covered by clayey tills and the topographic gradient is diminished. Maximum annual discharge increases from <1 m^3^/s at site 1 to >80 m^3^/s at site 23. Below the largest WWTP, nighttime hypoxia is a stressor because river DO varies from less than 1 mg/L on hot summer nights to over 14 mg/L during the day.

Three sampling campaigns were designed to capture the effects of seasonal variability during lower flows and non-ice covered seasons when biological transformation should be the most effective: 14-June-2007 (early summer, peak photosynthetic activity), 5-September-2007 (late summer, end of growing season, past peak biomass) and 24-April-2009 (spring, higher flows and prior to the emergence of macrophytes). Samples were collected close to solar noon on the specified dates by teams of samplers who waded into the river to manually collect the samples or sampled from piers or bridges in the lower river sections. Travel time of the river from one sampling site to the next was not explicitly factored into the sampling design as sites were sampled at approximately the same time of the day (within 2 hours). Samples were kept on ice in the dark and filtered to 0.45 µm in the laboratory before being stored frozen until analysis. Stability data for samples processed to date shows no indication that freezing causes the four AS to precipitate out of solution (data not shown).

Effluent discharge volume from the larger WWTPs exhibits daily fluctuations (a factor of 2.5) with peaks in morning and evening corresponding to the beginning and end of a normal workday. As a result, AS concentrations downstream from the WWTPs also vary on a daily cycle with changes in the dilution factor. Samples were collected at 6 to 8 sites within the effluent plume from below each of the two largest WWTPs (Kitchener, Waterloo) to a distance of 5 km in 2 different years. The plume location and highest concentrations were independently confirmed using conductivity, chloride and bromide. Results demonstrate the conservative nature of AS in the WWTP plume in the river at this distance (data not shown). Samples were also collected within the two largest WWTPs from the effluent stream every 3 hours on a diel basis on 4 occasions. Plume and WWTP samples were processed as above.

Municipal water supply samples were collected from private residences at taps not influenced by additional treatment systems (e.g. water softeners). Prepared sample containers were filled and stored frozen prior to transport to the laboratory for processing and analysis.

The four sweeteners were analyzed by ion chromatography (Dionex 2500 system) coupled with tandem mass spectrometry (AB Sciex QTRAP 5500 triple-quadrupole), operated in negative electrospray ionization (ESI) mode. Sample pre-treatment (e.g. SPE) is not required for this method, thereby increasing sample throughput compared to some other methods used for AS analysis. The minimum detection limits (mdl) for acesulfame, saccharin, cyclamate, and sucralose were 0.008, 0.021, 0.003, and 5 µg/L, respectively. Precision for the method is better than ±20% for all four artificial sweeteners. For sucralose, our mdl and practical quantification limit (pql: 15 µg/L) are relatively high compared to other analytical methods (e.g. 0.01 µg/L [3]) and therefore a detailed analysis of our sucralose data in the Grand River was not included here. A comprehensive description of the analytical methods has been published in the supplementary material (Appendix A) of Van Stempvoort et al. [4].

The field work conducted for this study did not involve endangered or protected species.

## Results and Discussion

**Figure 2:**
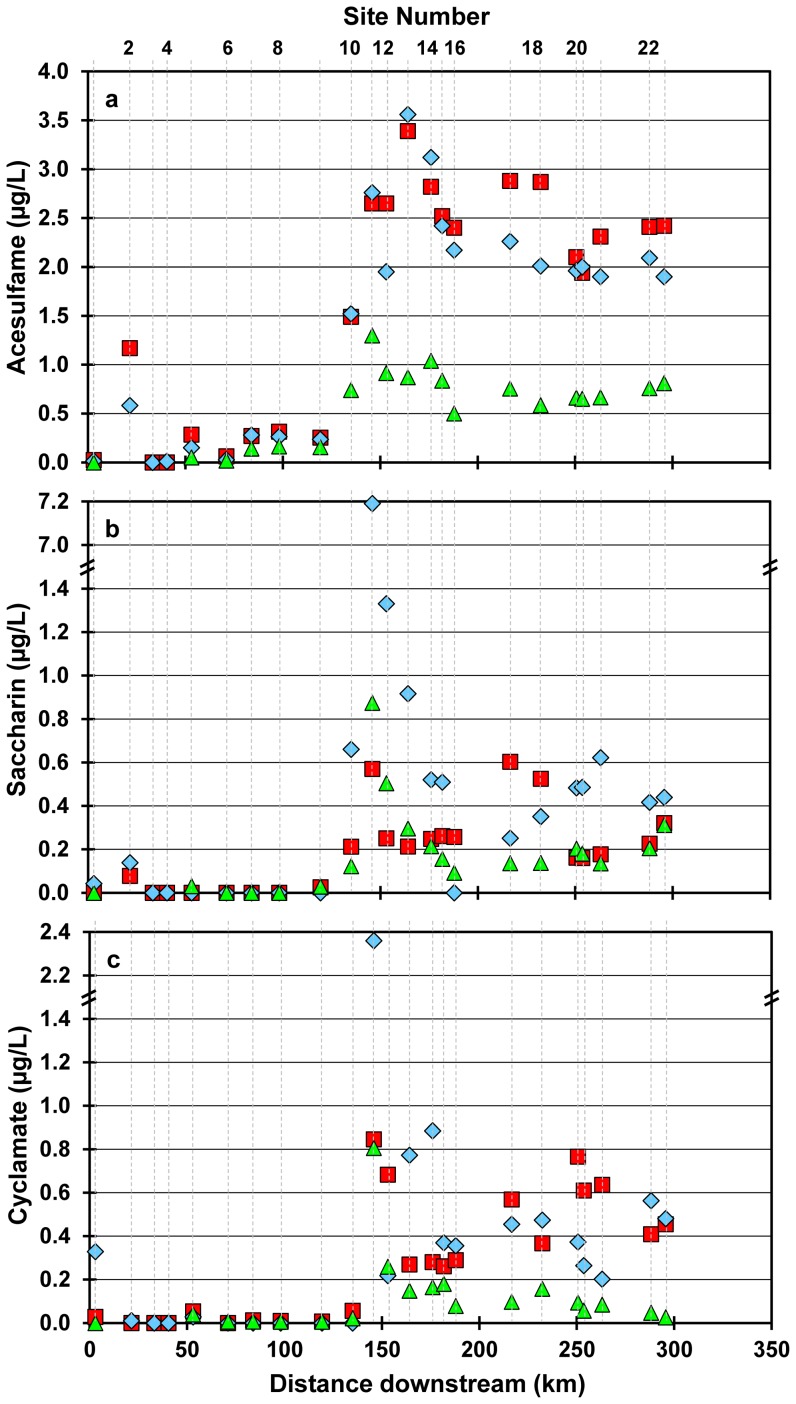
Concentrations of 3 artificial sweeteners in the Grand River. Acesulfame (a), saccharin (b) and cyclamate (c) concentrations in the Grand River on three sampling dates; Jun 2007 (blue diamonds), Sep 2007 (red squares), Apr 2009 (green triangles). Samples plotted at y  =  “0” have concentrations less than the minimum detection limit.

**Figure 3:**
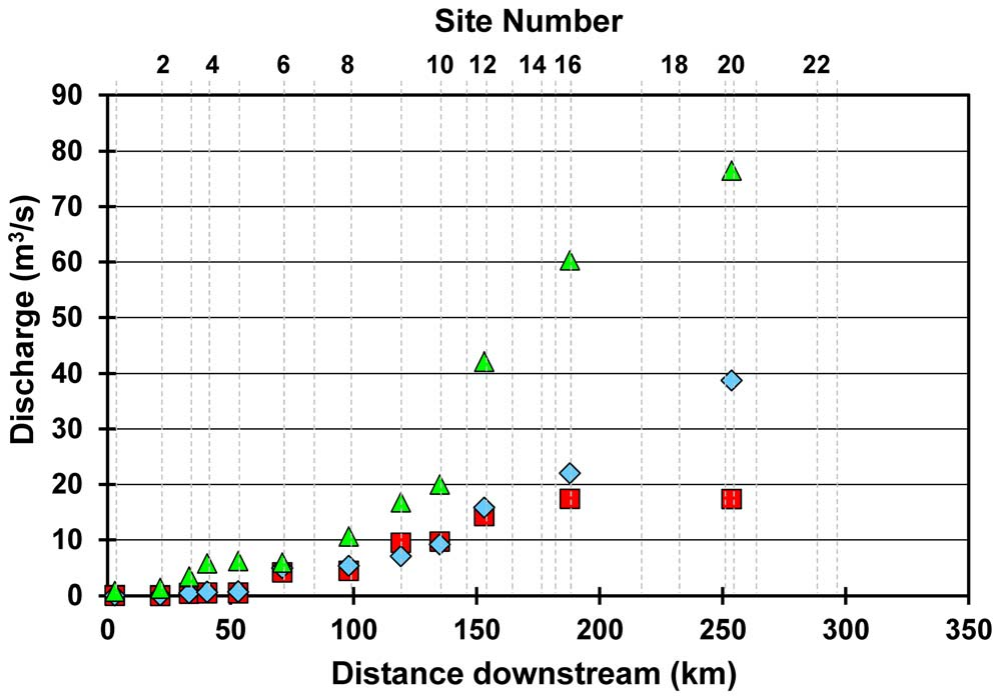
Grand River discharge. Daily mean discharge for the Grand River at gauged sites for each sampling date; Jun 2007 (blue diamonds), Sep 2007 (red squares), Apr 2009 (green triangles).

All four AS analyzed were detected at elevated concentrations in the Grand River (Fig. 2, Table 1). The larger river volume available to dilute WWTP effluent resulted in lower AS concentrations during April compared to June or September (Fig. 3). The maximum concentrations that we measured for sucralose (21 µg/L), cyclamate (0.88 µg/L), and saccharin (7.2 µg/L) are the highest reported concentrations of these compounds in surface waters to date anywhere in the world.

**Table 1:** Summary of published data on the concentration of artificial sweeteners measured in freshwater surface waters (streams and lakes) and the data from the Grand River.

			Concentration (µg/L)			
Reference	Country	n	Acesulfame	Saccharin	Cyclamate	Sucralose
[1]	Sweden	15				<mdl to 3.56
[3]	Germany	23	0.27 to 2.7	0.01 to 0.35	0.03 to 0.32	0.01 to 0.11
[4]	Canada	9	<mdl to 0.34	<mdl to 0.066	<mdl	<mdl
[8]	Germany	3–4	2.0*	0.01*	0.25*	0.05*
[9]	Germany	24	2.1 to 3.6	0.03 to 0.11	0.10 to 0.24	0.12 to 0.16
[10]	USA					<mdl to 2.9
[13]	USA	22		<mdl		<mdl to 1.8
[14]	USA	26				<mdl to 10
[27]	Switzerland	20	<mdl to 6.9	<mdl to 0.18	<mdl to 0.13	<mdl to 0.6
[28]	EU	125				<mdl to 0.924
[29]	Germany	1	23†			
[30]	Sweden	3				0.11 to 0.41
[31]	Switzerland	80	<mdl to ∼10			
This study	Canada	57	<mdl to 3.6	<mdl to 7.2	<mdl to 0.88	<mdl to 21

n  =  the number of samples; does not include our measurements in the WWTP plume.

<mdl  =  less than the minimum detection limit.

Blank cells indicate that the parameter was not reported.

* Maximum value reported.

† About 50% of flow is derived from wastewater sources.

Saccharin and cyclamate concentrations in the Grand River ranged from less than the minimum detection limit (<mdl) to 7.2 and 0.88 µg/L, respectively. Many of these values, measured downstream of the major urban centres (e.g. below site 9), were higher than previously reported for rivers (Table 1). Although previously detected in groundwater [4]–[7], our study is the first to report detectable cyclamate concentrations for a North American river.

Sucralose had the highest concentration of any of the artificial sweeteners (max. value of 21 µg/L). However, only two samples in the synoptic survey had sucralose concentrations above our quantifiable limit, both at a relatively short distance (23.8 to 35.8 km) below the largest WWTP. Sucralose has previously been measured at concentrations ranging from <mdl to 3.56 µg/L in rivers in European countries [1]–[3], [9] and <mdl to 10 µg/L in North America [4], [10], [13], [14].

Acesulfame was the most consistently detected AS and present at 21 of the 23 sites. In addition to contributions of much smaller WWTPs along the river and tributaries, acesulfame is the only one of the 4 AS to record the input from WWTP lagoons located just upstream of site 2. Concentrations reached as high as 3.6 µg/L downstream of the main urban centre, comparable to levels reported for European surface waters (<mdl to 6.9 µg/L, Table 1) and an order of magnitude higher than previously reported for Canada (<mdl to 0.34 µg/L, Table 1). Acesulfame was often the only AS detected in the upper reaches of the watershed.

Cyclamate and saccharin are more easily degraded during WWTP processes whereas removal rates for acesulfame and sucralose are very low to not detectable [1], [3]. As a result, cyclamate and saccharin concentrations in WWTP effluents and receiving waters are typically much lower than acesulfame and sucralose [2], [3], (this study). Acesulfame and sucralose have been proposed as tracers of wastewater in aquatic systems because of their conservative nature and ubiquitous occurrence [2], [3]. We show here that all 4 of these AS pass through WWTPs resulting in elevated concentrations in the Grand River (Fig. 2). Unusually high concentrations of saccharin and cyclamate, recorded at site 11 in June 2007, could be the result of the discharge of under-treated wastewater from the largest WWTP. Effluent samples collected within this WWTP in June 2007 also show unusually high saccharin and cyclamate concentrations compared to effluent samples collected on 3 other dates (data not shown). High saccharin and cyclamate concentrations in the effluent likely resulted from a decreased hydraulic retention time within the WWTP, although the specific mechanism responsible is not known.

**Figure 4:**
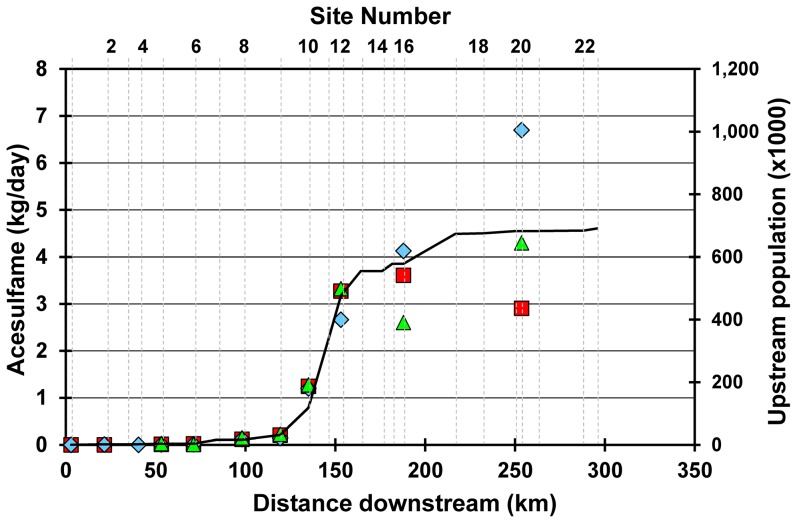
Mass flux of acesulfame in the Grand River. Daily mass flux of acesulfame at gauged sites for each sampling date; Jun 2007 (blue diamonds), Sep 2007 (red squares), Apr 2009 (green triangles). Cumulative population contributing to WWTP discharge upstream of each site is indicated by the black line.

Relatively little is known about the fate and effects of artificial sweeteners in rivers. Acesulfame has been shown to behave conservatively in groundwater [2], [5], [6] indicating that biogeochemical activity in the subsurface does not significantly affect acesulfame. In contrast, sucralose is attenuated under aerobic and sub-oxic to anoxic conditions [3], [5], [6], [15], [16]. Our samples collected in the plumes below two of the largest WWTPs to a distance of 5 km also demonstrate the persistent nature of all four AS in temperate rivers despite the large range in daily DO and high rates of microbiological activity. Furthermore, elevated concentrations of acesulfame persist for over 300 km in the Grand River and reflect the cumulative human population in the watershed (Fig. 4). Because of the daily variation in discharge at the large WWTPs (a factor of 2.5), single river samples may not capture the full diel range of AS concentrations, especially close to the WWTPs where the river is not fully mixed. However, the general agreement of increasing acesulfame with population (Fig. 4.) demonstrates the conservative behavior of acesulfame in natural waters and its suitability as a long-term tracer of wastewater in the environment.

Large rivers often serve as raw water sources for municipal potable water production. Acesulfame is also only partially removed by the various processes used in municipal water treatment plants. Of the common treatment methods used, ozonation is the most effective but complete removal of acesulfame is unlikely given ozone concentrations and treatment times typically used in water treatment plants [9]. Acesulfame is therefore the most suitable of the four AS as a tracer of wastewater contamination from source water to end user.

In addition to receiving the effluent from 30 WWTPs, the Grand River is a source of raw water for drinking water production for major urban centres in the watershed. For example, Brantford takes 100% of its municipal water from the Grand River. Kitchener and Cambridge receive groundwater supplemented by Grand River water via an artificial recharge scheme. In contrast, Waterloo typically receives only groundwater as a municipal water source. Given the high concentrations of AS in the Grand River, especially downstream of the City of Waterloo, it is not surprising that AS were also detected in tap water in these large cities (Table 2). Brantford had the highest concentrations of AS of the cities sampled, including high concentrations of more easily degraded saccharin. Brantford’s water treatment system consists of screening, coagulation, sand ballasted flocculation, sedimentation, ozonation, biological filtration, UV disinfection, chlorination and chloramination [17].

**Table 2:** Concentration of artificial sweeteners in municipal water collected from household taps.

		Concentration (µg/L)		
Water Source	n	acesulfame	saccharin	cyclamate
Brantford: river water	7	0.55 – 1.59	0.18 – 0.35	<mdl (6), 0.24
Cambridge: groundwater, groundwater from ARS	9	<mdl (4), <pql (3), 0.04 – 0.39	<mdl (7), <pql (2)	<mdl (8), 0.01
Kitchener: groundwater, groundwater from ARS	4	0.08 – 0.12	<pql (3), 0.07	<mdl (4)
Waterloo: groundwater	8	0.05 – 0.12	<mdl (2), <pql (6),	<mdl (8)

n  =  total number of samples.

Brackets indicate the number of each type of result.

<mdl  =  less than the minimum detection limit.

<pql  =  below the practical quantification limit.

ARS  =  artificial recharge scheme.

Artificial sweeteners in municipal tap water could also result from the presence of groundwater that has been impacted by leaking sewer pipes (e.g. [8]) that subsequently enters compromised water supply mains. For areas sourcing their water from groundwater aquifers, septic tile bed plumes are another source of AS [5], [6]. The presence of acesulfame in municipal water distribution systems could be a very sensitive way of detecting areas where old or failing infrastructure has compromised the integrity of the sewer and water systems.

Although concentrations of AS in the Grand River are small compared to the products they are derived from (e.g. diet drinks), mass fluxes of these compounds to Lake Erie via the Grand River are substantial. The mass of acesulfame flowing past site 20 ranged from 2.9 to 6.7 kg/day (Fig. 4) or 4 to 10 mg/person/day, similar to the WWTP effluent loading of acesulfame in the region of Zurich, Switzerland (11±4.2 mg/person/day [2]). Calorie reduced beverages (mainly carbonated soft drinks) constitute a major contributor to the human dietary intake of AS, including acesulfame [13]. Given a mean acesulfame concentration of about 100 mg/L in these beverages [18]–[21], this mass flux equates to the equivalent amount of acesulfame in 81,850 to 188,650 355mL-cans of soda pop flowing past site 20 each day or 0.12 to 0.28 cans of soda pop per person.

Although some studies of the effects of sucralose on aquatic biota have been done [22]–[26], the ecological effects of AS on aquatic organisms are largely unknown. Furthermore, even less is known about the chemical breakdown products of AS in the aquatic environment or their toxicity. We demonstrate here that aquatic organisms likely experience long-term exposure to significant concentrations of AS downstream of urban centres that discharge WWTP effluents. Furthermore, impacts are not confined to the immediate reach below WWTPs but persist for hundreds of kilometers. In systems where both animal manure and human sewage are potential sources of contamination, presence or absence of acesulfame is a powerful geochemical tool to distinguish between these two sources. As we are not aware of acesulfame currently being used in animal feed, the presence of acesulfame indicates an anthropogenic wastewater source. Our finding that acesulfame loading reflects the human population in the Grand River Watershed, coupled with the fact that human sources are concentrated in the center of the watershed whereas large livestock operations are distributed throughout the watershed, supports the use of acesulfame to separate animal and human waste sources. In contrast, saccharin is used in animal feed [27], primarily for pigs. Therefore, in the absence of acesulfame, saccharin in groundwater or surface water could indicate a nearby animal manure source, likely of porcine origin. Since cyclamate and saccharin are largely degraded in WWTPs or by natural processes in groundwater and surface water, presence of these compounds at high concentrations signifies recent contamination by under-treated sewage or a proximal source. Thus the combination of different AS can also be used to trace and differentiate human and animal waste sources.

At the larger scale, Lake Erie receives discharge from numerous rivers like the Grand River from both Canada and the United States. Given that AS have been in use for many decades and both use of AS and population have been increasing, AS concentrations in the Great Lakes, including Lake Erie, are likely increasing. Acesulfame could be used as a tracer of wastewater impact at the scale of the Great Lakes, especially in the nearshore environment.

## Conclusions

Our study demonstrates elevated levels of AS in a large, human-impacted river. The ubiquitous occurrence of acesulfame in wastewater effluents, its high concentration coupled with the high sensitivity of available analytical methods, and its resistance to breakdown in both WWTPs and in groundwater and surface water environments, makes it an ideal tracer of human derived wastewater. Acesulfame will be particularly useful for studying groundwater - surface water interaction, nutrient assimilation and other wastewater constituents including emerging contaminants released to rivers, lakes, and nearshore marine environments. Acesulfame can be used to distinguish and quantify dilution versus attenuation and it circumvents problems of confounding source inputs common with other tracers such as chloride (e.g. road salt, groundwater inputs). Given the persistent nature demonstrated here and solely human source, we expect that acesulfame will become the most reliable detector of wastewater presence, dilution, and transformation in surface and ground waters.
