# Correction: Artificial Sweeteners in a Large Canadian River Reflect Human Consumption in the Watershed

**DOI:** 10.1371/journal.pone.0106394

**Published:** 2014-08-22

**Authors:** The PLOS ONE Staff

The maximum value for the concentration of cyclamate appears incorrectly throughout the paper as 0.88 µg/L. The correct maximum value for the concentration of cyclamate is 2.4 µg/L. This error appears in the Abstract, the first and second paragraph of the Results and Discussion and the last row of Table 1.
